# miRNAs as radio‐response biomarkers for breast cancer stem cells

**DOI:** 10.1002/1878-0261.12635

**Published:** 2020-02-06

**Authors:** Carmen Griñán‐Lisón, María Auxiliadora Olivares‐Urbano, Gema Jiménez, Elena López‐Ruiz, Coral del Val, Cynthia Morata‐Tarifa, José Manuel Entrena, Amanda Rocío González‐Ramírez, Houria Boulaiz, Mercedes Zurita Herrera, María Isabel Núñez, Juan Antonio Marchal

**Affiliations:** ^1^ Biopathology and Regenerative Medicine Institute (IBIMER) Centre for Biomedical Research (CIBM) University of Granada Granada Spain; ^2^ Instituto de Investigación Biosanitaria ibs.GRANADA Spain; ^3^ Department of Radiology and Physical Medicine University of Granada Spain; ^4^ Bio‐Health Research Foundation of Eastern Andalusia ‐ Alejandro Otero (FIBAO) Granada Spain; ^5^ Department of Health Sciences University of Jaén Spain; ^6^ Department of Artificial Intelligence University of Granada Spain; ^7^ Andalusian Network for Design and Translation of Advanced Therapies Sevilla Spain; ^8^ Department of Human Anatomy and Embryology Faculty of Medicine University of Granada Spain; ^9^ Radiation Oncology Department Virgen de las Nieves University Hospital Granada Spain

**Keywords:** biomarkers, breast cancer, CSCs, miRNAs, radiation, radiotherapy

## Abstract

In breast cancer (BC), the presence of cancer stem cells (CSCs) has been related to relapse, metastasis, and radioresistance. Radiotherapy (RT) is an extended BC treatment, but is not always effective. CSCs have several mechanisms of radioresistance in place, and some miRNAs are involved in the cellular response to ionizing radiation (IR). Here, we studied how IR affects the expression of miRNAs related to stemness in different molecular BC subtypes. Exposition of BC cells to radiation doses of 2, 4, or 6 Gy affected their phenotype, functional characteristics, pluripotency gene expression, and *in vivo* tumorigenic capacity. This held true for various molecular subtypes of BC cells (classified by ER, PR and HER‐2 status), and for BC cells either plated in monolayer, or being in suspension as mammospheres. However, the effect of IR on the expression of eight stemness‐ and radioresistance‐related miRNAs (miR‐210, miR‐10b, miR‐182, miR‐142, miR‐221, miR‐21, miR‐93, miR‐15b) varied, depending on cell line subpopulation and clinicopathological features of BC patients. Therefore, clinicopathological features and, potentially also, chemotherapy regimen should be both taken into consideration, for determining a potential miRNA signature by liquid biopsy in BC patients treated with RT. Personalized and precision RT dosage regimes could improve the prognosis, treatment, and survival of BC patients.

## Graphical abstract

Breast cancer stem cells (BCSCs) have been related to relapse, metastasis, self‐renewal, and radioresistance. Ionizing radiation (IR) modifies miRNA expression. miRNAs related with BCSCs, tumorigenic process, radioresistance and poor prognosis showed variability in the expression according to IR dose, BC molecular subtypes and clinicopathological features. This miRNA signature could improve prognosis, treatments, and survival of BC patients by using precision radiotherapy dosage regimes.

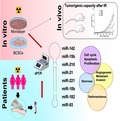

**Abbreviations**
ALDH1: aldehyde dehydrogenase 1
BC: breast cancer
BCSC: breast cancer stem cell
CSC: cancer stem cell
EMT: epithelial‐to‐mesenchymal transition
IR: ionizing radiation
RT: radiotherapy
TNBC: triple‐negative breast cancer

## 1 Introduction

Breast cancer (BC) is the second most common cancer in the world and, by far, the most frequent cancer among women. Despite advances made in current treatments against BC such as surgery, chemotherapy, radiotherapy (RT), and immunotherapy, different studies showed that 30–50% of patients will develop metastasis (Gangopadhyay *et al.*, 2013; Qi *et al.*, 2017). This is probably due, among other factors, to the cancer stem cells (CSCs), a small subpopulation of cancer cells with reduced proliferative potential but with the ability of self‐renewal and tumorigenicity (Al‐Hajj and Clarke, 2003; Cojoc *et al.*, 2015). They provide the key to unlocking new insights into the mechanisms driving BC progression, drug and radioresistance and metastasis (Owens and Naylor, 2013).

In the last decade, regarding CSC subpopulations in primary human BC, ESA^+^/CD44^+^/CD24^−/low^ phenotype has been used extensively to identify and isolate BC stem cells (BCSCs) with increased tumorigenicity (Ablett *et al.*, 2012). Combining these markers with aldehyde dehydrogenase 1 (ALDH1) activity that is increased in BCSCs, this fraction was refined further compared to only using either method alone (Owens and Naylor, 2013).

Cancer stem cells have been found to exhibit a number of genetic and cellular adaptations that confer resistance to RT. Among others, efficient DNA repair, the role of the CSC microenvironment and hypoxia (Brunner *et al.*, 2012), and the resistance to apoptosis through the activation of the Akt pathway (Morrison *et al.*, 2011) should be considered. The cell cycle phase also determines radiosensitivity, with cells being most radiosensitive in the G2‐M phase (Pawlik and Keyomarsi, 2004). Cells repair sub‐lethal damage between irradiation fractions, and, therefore, a failure of radiation treatment might be attributed to the incomplete eradication of CSC subpopulations (Krause *et al.*, 2011; Pawlik and Keyomarsi, 2004). Furthermore, it has been well established that miRNAs play a crucial role in the cellular response to ionizing radiation (IR) (Peitzsch *et al.*, 2013). It is a small endogenous non‐coding RNA molecule that regulates gene expression (Feinbaum *et al.*, 2004). Hence, miRNA expression changes could be useful for monitoring exposures and understanding regulation in response to radiation‐induced DNA damage (Cellini *et al.*, 2014; Czochor and Glazer, 2014); for example, studies have shown that miR‐125b and miR‐139 could be useful biomarkers of radiosensitivity (Metheetrairut *et al.*, 2017; Pajic *et al.*, 2018). Alike, it has been observed that the overexpression of miR‐26‐5p after radiation exposure is related to processes of breast carcinogenesis or that the miR‐223 expression (a potential tumor suppressor) in BC patients after radiation treatment restrains recurrence formation *via* EGF/EGFR pathway (Fabris *et al.*, 2016; Wilke *et al.*, 2018). For these reasons, miRNAs could be good cancer biomarkers for diagnostic, prognostic, and treatment response that could improve the efficacy of current cancer therapy (Halvorsen *et al.*, 2017; Schwarzenbacher *et al.*, 2013).

In this work, we have analyzed the effect of several IR doses (2, 4, and 6 Gy) on different molecular subtypes of BC cell lines (according to ER, PR, and HER‐2 status) and on their corresponding BCSC subpopulations. Moreover, we evaluated *in vitro* and in BC patients’ serum how IR affects the expression of a set of miRNAs selected from bibliographic sources using key words like ‘IR and miRNAs’, ‘miRNAS and IR and BC’, ‘miRNAs and BC’, ‘miRNAs and CSCs’, and ‘miRNAs and BCSCs and IR’. Thus, we have selected a set of miR, such as miR‐21, miR‐221, miR‐182, miR‐210, miR‐93, miR‐142, miR‐10b, and miR‐15b that are related to radioresistance, stemness properties, DNA repair, and metástasis in order to test their usefulness as biomarkers in the clinical arena, particularly in radiation oncology to predict and monitor tumor radio‐response.

## 2 Material and methods

### 2.1 Description of selection criteria and filter process

The steps followed for the selection of miRNAs were as follows:

- Generate programmatically a list of publications related to the topic using the search terms: ‘[ionizing radiation AND miRNAs]’, ‘[miRNAS AND ionizing radiation AND BC]’, ‘[miRNAs and BC]’, ‘[miRNAs AND CSCs]’ and ‘[miRNAs and BCSCs and ionizing radiation]’ through the Entrez Direct (Kans, 2010) unix access to NCBI’s suite of databases.
- Then, we generated a script that searched in each title and abstract selected for joint occurrences of biological processes (see underneath) and miRNA gene names.
- The resulting list was further analyzed with another script that gave to each miRNA a relevant score depending on the number and type of biological processes that appeared to be related to. The list of biological processes used was as follows: DNA damage repair (DDR), hypoxia, apoptosis, cell cycle, metastasis, invasion, and proliferation.

The resulting list contained 10 miRNAs, the ones presented in this study plus miR‐34a and miR‐125b. However, after weak preliminary *in vitro* results (data not shown) we decided to discard these miRNAs for further analysis.

### 2.2 Cell lines

The three human BC cell lines MCF7 (ER^+^, PR^+^, HER2^−^), MDA‐MB‐231 (ER^−^, PR^−^, HER2^−^), and SKBR3 (HER2+) were obtained from American Type Culture Collection (ATCC, Manassas, VA, USA) and maintained in Dulbecco’s Modified Eagle Medium (DMEM; Sigma‐Aldrich, St Louis, MO, USA) supplemented with 10% FBS (BioWhittaker; Lonza, Basel, Switzerland) and with 1% of a solution of penicillin/streptomycin (10 000 U·mL^−1^ penicillin G and 10 mg·mL^−1^ streptomycin; Sigma‐Aldrich).

### 2.3 Isolation, enrichment, and characterization of BCSCs

Breast cancer stem cells were isolated by fluorescence‐activated cell sorting (FACS Aria, BD Biosciences, San Jose, CA, USA) using the ALDEFLUOR assay (Stem Cell Technologies, Vancouver, Canada) according to the manufacturer's instructions. For ALDH1 + CSCs culture, mammospheres were maintained in sphere medium (DMEM‐F12; Sigma‐Aldrich), 1% streptomycin/penicillin (Sigma‐Aldrich), 1 mg·mL^−1^ hydrocortisone (Sigma‐Aldrich), 4 ng·mL^−1^ heparin (Sigma‐Aldrich), 1× ITS (Gibco, Big Cavin, OK, USA), 1× B27 (Gibco), 10 ng·mL^−1^ EGF (Sigma‐Aldrich), and 10 ng·mL^−1^ FGF (Sigma‐Aldrich) in ultra‐low attachment plates (Corning Inc., Corning, NY, USA).

Cell surface marker levels of CSCs were determined with human antibodies anti‐CD44‐PE and anti‐CD24‐APC (Miltenyi Biotec, Auburn, CA, USA) and ALDEFLUOR assay (Stem Cell Technologies) to detect enzyme ALDH1 activity was performed to complete characterization (Li *et al.*, 2017; Rabinovich *et al.*, 2018; Wang *et al.*, 2017). Samples were measured and analyzed by flow cytometry on a FACS CANTO II (BD Biosciences, San Jose, CA, USA).

### 2.4 Cell radiation protocol

Attached cells and BCSC suspension were irradiated by the X‐ray equipment Yxlon Smart Maxishot 200‐E at room temperature, under a constant current of 4.5 mA and power of 200 kW at different doses of 2 Gray (Gy), 4 Gy, and 6 Gy, and cultured for 24 h. Sham‐irradiated cells were used as control (0 Gy). For the field size of 15 cm × 8 cm, the focal distance was 15 cm, and for 11.3 cm × 7 cm field size, focal distance was 25 cm. Traceable dosimetry was performed following protocol TRS.398.

### 2.5 Secondary mammosphere‐forming and soft agar assay

For the secondary mammosphere‐forming assay, cells from primary mammospheres irradiated 24 h before at 2, 4, and 6 Gy were collected by centrifugation, then dissociated with trypsin‐EDTA, and mechanically disrupted with a pipette. Cells from sham‐irradiated 0 Gy primary mammospheres were used as control. One thousand to Two thousand single cells (depending on the cell line plating efficiency) were plated and resuspended in spheres culture medium in ultra‐low adherence 24‐well plates. Spheres were counted after 5 days by light microscopy.

For colonies' formation, ALDH1 + mammospheres 0 Gy control and irradiated at 2, 4, and 6 Gy were disaggregated and seeded in 0.4% cell agar base layer (1 × 10^4^ cells), which was on top of 0.8% base agar layer in 6‐well culture plates after 24 h. Cells were then incubated for further 28 days at 37 °C and 5% CO_2_. Cell colony formation was then counted under a light microscope after staining with 1 mg·mL^−1^ iodonitrotetrazolium chloride (Sigma‐Aldrich) overnight at 37 °C.

### 2.6 Functional annotation of miRNAs

We data mined relevant existing literature about the eight selected miRNAs in PubMed (https://www.ncbi.nlm.nih.gov/pubmed/) through the Entrez Direct (Kans, 2010) unix access to NCBI’s suite of databases. The search was narrowed down to the last 10 years. The articles retrieved were manually inspected, and miRNA functions were categorized according to known Cancer Hallmarks (Hanahan and Weinberg, 2011), radioresistance, and stemness. The obtained data were completed using pathway (Fabregat *et al.*, 2017; Kanehisa and Goto, 2000) and Gene Ontology (Carbon *et al.*, 2017) annotation for the studied miRNAs. The resulting data were analyzed using clustering, an unsupervised learning technique common for statistical data analysis, to group the obtained functional data into a specific group with similar properties and/or features. Analysis was performed using the Cluster Analysis Basics and Extensions for the r language (Maechler *et al.*, 2019).

### 2.7 Quantitative real‐time‐PCR

Total RNA from different cell lines was extracted from both 80% confluent adherent cell and ALDH1 + mammospheres after 5 days of culture in cell suspension, using the TRIZOL reagent following the manufacturer’s instructions (Sigma‐Aldrich). cDNA was synthesized by reverse transcription of total RNA using the Reverse Transcription System (Promega, Madison, WI, USA) for mRNA, and miRCURY LNA TM Synthesis kit II (Exiqon, Vedbaek, Denmark) for miRNAs. Quantitative real‐time‐PCR (qRT‐PCR) assay was done using SYBR Green PCR Master Mix (Promega) and miRCURY LNA TM EXILENT SYBR Green (Exiqon) for miRNAs. Each experiment was done in duplicate, and reactions were performed in triplicate. The comparative threshold cycle (Ct) method was used to calculate the amplification factor as specified by the manufacturer ABI 7500. For mRNAs, human GAPDH was used as an internal standard to normalize and hsa‐miR‐24‐3p, RNU6, and hsa‐miR‐425‐5p for miRNAs. The amount of target and endogenous reference was determined from a standard curve for each experimental sample. Primer sequences are listed in Table S1 (mRNAs) and Table S2 (miRNAs).

### 2.8 *In vivo* tumor orthotopic xenograft assays

Tumor initiation ability assays into mammary fat pads were done using both monolayer at 80% confluence and mammosphere MDA‐MB‐231 [triple‐negative breast cancer (TNBC)] after 24 h of irradiation at 2, 4, and 6 Gy and a 0 Gy control. Three thousand of each condition were injected in 0.05 mL matrigel and 0.05 mL culture medium into one inguinal mammary fat pad of 8‐week‐old NOD scid mice gamma (NOD.Cg‐Prkdcscid Il2rgtm1Wjl/SzJ, NSG). Tumor growth was assessed twice weekly using a digital caliper, and the tumor volume was calculated by the formula *V* = length^2^ × width × π/6. Animal experimentation was performed according to the protocols reviewed and approved by the Institutional Animal Care and Use Committee of the University of Granada (PI730/13).

### 2.9 Histological and immunofluorescence analysis

Tumors of different conditions were fixed in 4% paraformaldehyde in 0.1 m PBS at 4 °C for 24 h, washed in 0.1 m PBS, and dipped in paraffin in an automatic tissue processor (TP1020; Leica, Germany). Paraffin blocks were cut into 4 mm, and sections were deparaffinized with xylene and hydrated with decreasing alcohol concentrations, and stained with hematoxylin‐eosin. Later, sections were dehydrated with increasing alcohol concentrations and were cleared with xylene. The stained slides were mounted on coverslips with mounting medium. Observation samples and digital image acquisition was carried out with an inverted microscope (Nikon H550s, Tokyo, Japan).

Then, for intracellular staining, sections were permeabilized with 0.1 % Triton X‐100 for 15 min, blocked for 1 h at room temperature with 5% BSA, 5% FBS in PBS, and incubated with the primary antibody overnight at 4 °C. For immunofluorescence analysis, primary antibodies used were purchased from Vimentin Santa Cruz Biotechnology (Dallas, TX, USA). Next day, samples were washed thrice with PBS and incubated with the secondary antibodies (Alexa, Waltham, MA, USA) for 1h at RT, after washing thrice with PBS and mounted with DAPI‐containing mounting medium. Images were taken by confocal microscopy (Nikon Eclipse Ti‐E A1, Tokyo, Japan) and analyzed using NIS‐Elements software. Its immunofluorescence intensity was qualified using imagej ^TM^ software (National Institutes of Health, Bethesda, MD, USA).

### 2.10 Breast cancer patients

Blood serum samples obtained from 20 women with BC were collected and analyzed for miRNA detection using q‐PCR. These patients were treated with either hypofractionated RT (16 fractions, 2.65 Gy/fraction) or conventional RT (25 fractions, 2 Gy/fraction). Three blood samples were collected from each patient at different times of the treatment, obtaining a total of 60 samples. First samples were taken approximately 1 week before the start of the RT; second samples were taken during the RT (depending on RT regimen received, 8 or 11 days after the start of the treatment); and third samples were taken on the last day of treatment. Written informed consent was obtained from all the patients in compliance with the Declaration of Helsinki. This study was approved by the corresponding ethical committee associated with grants PI‐730 and PIE16‐00045.

### 2.11 Statistical analysis

All statistical tests were performed with the statistical Package for the IBM‐spss Statistics Ver.21.0. (IBM Corp., Armonk, NY, USA) Variables with normal distribution were expressed as mean ± SEM. For quantitative variables, when two groups were compared, we used Student’s *t*‐test (parametric) in a case of normality or Mann–Whitney *U* test (nonparametric) for non‐normal. For comparisons between multiple means, nonparametric tests of Kruskal–Wallis were used. Differences were considered statistically significant at *P* < 0.05 level.

Data charts were carried out using Microsoft® Excel (Microsoft Corporation, Redmond, WA, USA) and r Statistical Computing Environment 3.4.0 (Lucent Technologies, Murray Hill, NJ, USA).

## 3 Results

### 3.1 Effects of IR on stemness properties

**Figure 1:**
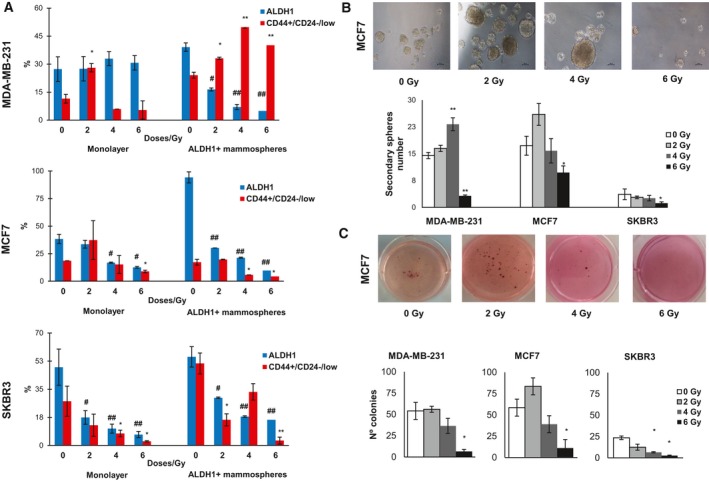
Phenotypic properties of monolayer and mammosphere cultures irradiated with different doses. (A) Variation of percentage of ALDH1 and CD44^+^/CD24^−/low^ in monolayer and mammosphere cultures. (B) Representative images of MCF7 mammospheres formed from different IR doses and number of spheres of each cell line. Scale bar = 100 μm (C) Representative images of MCF7 colonies formed from different IR doses and the number of colony‐forming ability of BC cell lines. Data are graphed as mean ± SEM (^#^ *P* < 0.05 or ^##^ *P* < 0.01 for ALDH1 expression; **P < 0.01; *P < 0.05).

Cells were characterized using, specific breast CSC characteristics, ALDH1 activity and CD44^+^/CD24^−/low^ expression, and results were compared with sham‐irradiated control cells (Fig. 1A). In MDA‐MB‐231 monolayer, ALDH1 activity was similar at different IR doses, while CD44^+^/CD24^−/low^ expression was significantly higher in 2 Gy (*P < 0.05). ALDH1 activity in mammospheres significantly decreased in all doses showing 4 Gy and 6 Gy had significantly lower ALDH1 activity (^##^ *P* < 0.01) in comparison with 2 Gy (*P < 0.05); however, CD44^+^/CD24^−/low^ expression was higher in all IR doses, being more significant in 4 and 6 Gy.

In MCF7 monolayer cells, ALDH1 activity and CD44^+^/CD24^−/low^ expression were significantly decreased at 4 and 6 Gy doses (^#^ *P* < 0.05, *P < 0.05), and increased at 2 Gy. On the other hand, mammospheres showed lower ALDH1 activity (^##^ *P* < 0.01) in all different IR doses, and we also observed in CD44^+^/CD24^−/low^ expression a similar behavior in that monolayer.

SKBR3 cell line showed an important decrease of ALDH1 activity in both subpopulations, which were very significant in all IR doses (^##^ *P* < 0.01) for 4 and 6 Gy; moreover, in monolayer CD44^+^/CD24^−/low^ expression was lower than the control in all IR doses, being more significant for 6 Gy (*P < 0.05), and in 2 and 6 Gy significantly decreased CD44^+^/CD24^−/low^ expression.

In general terms, the expression level of ALDH1 decreased with IR dose in the cell lines in both culture models. In contrast, the expression of CD44^+^/CD24^−^ increased with IR dose in MDA‐MB‐231.

Also, real‐time RT‐PCR analysis was used to quantify the effect of IR in the expression of specific transcription factors (*NANOG, SOX2,* and *OCT4*) that promote stemness properties, and those related to epithelial‐to‐mesenchymal transition (EMT) process (*E‐CADHERIN, N‐CADHERIN,* and *VIMENTIN*; Fig. S1). In MDA‐MB‐231 monolayer, 4 Gy produced an increment in the expression of *NANOG* and *SOX2*, and a significant *SOX2* and *OCT4* higher expression for 6 Gy (*P < 0.05) in ALDH1 + mammospheres. *N‐CADHERIN* expression showed an increase for 2 Gy in both monolayer and mammospheres. At 6 Gy, MCF7 monolayer cells displayed an increment in *NANOG, SOX2,* and *OCT4* genes’ expression. Related to EMT genes in monolayer cultures, *VIMENTIN* was overexpressed for 2 and 4 Gy (*P < 0.05) and *N‐CADHERIN* showed a higher increment for 4 and 6 Gy. Finally, SKBR3 cells grown in monolayer showed higher expression pluripotency genes for 4 Gy. Also, *VIMENTIN* showed higher expression for 4 and 6 Gy (*P < 0.05) in monolayer, and an *N‐CADHERIN* increase in mammospheres (**P < 0.01) for 2 Gy. These data suggests that, depending on IR doses and molecular profile, the stemness phenotype is differentially modulated.

### 3.2 Effects of IR on self‐renewal ability and clonogenicity over ALDH1 + mammospheres

To study the effect of IR doses on BCSC functional characteristics, both mammosphere formation and clonogenic capacity of ALDH1 + cells were analyzed. As is shown in Fig. 1B and Fig. S2A, the mammosphere number was higher at 4 Gy (**P < 0.01) in MDA‐MB‐231 cell line, and at 2 Gy in MCF7 cell line, compared to respective controls. In contrast, SKBR3 showed a minor mammosphere formation ability for all different IR doses. Interestingly, a 6 Gy dose significantly inhibited secondary mammosphere formation in MDA‐MB‐231 (**P < 0.01), MCF7 (*P < 0.05), and SKBR3 (*P < 0.05) cell lines. In concordance with these results, 6 Gy irradiated cells showed a lower capacity to form colonies in soft agar in comparison with 0 Gy cells (*P < 0.05). Also, 4 Gy significantly decreased clonogenicity in Her + BC cells (*P < 0.05; Fig. 1C and Fig. S2B). These data suggest that 2 Gy in MDA‐MB‐231 and MCF7 cell lines was the most efficient in maintaining stemness properties; however, SKBR3 cell line lost the majority of these properties when was irradiated.

### 3.3 *In vivo* monitoring effects of IR

**Figure 2:**
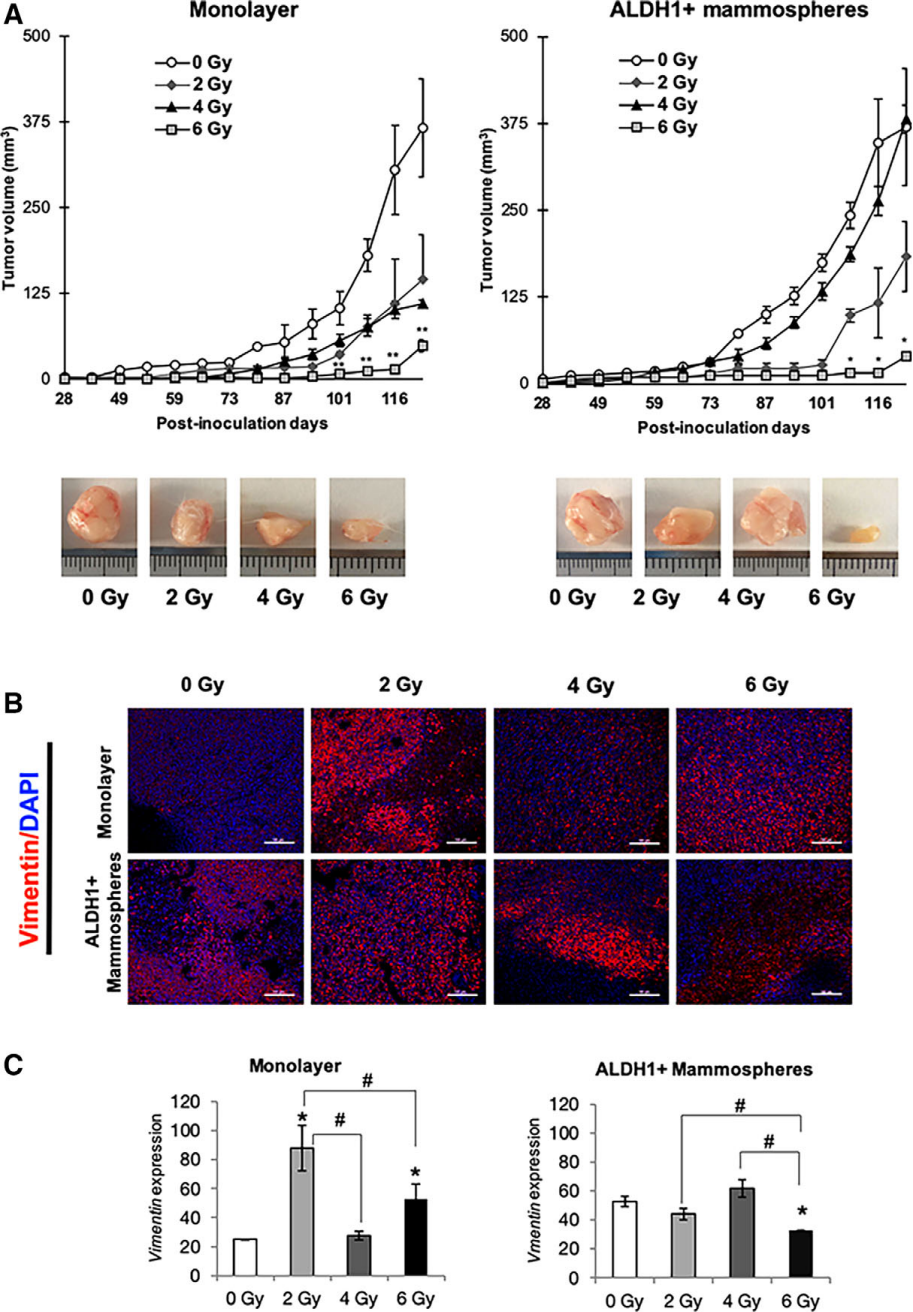
*In vivo* analysis of tumorigenic capacity of BC cell line MDA‐MB‐231 after irradiation. (A) Tumor volume of orthotopic xenograft mammary gland tumors formed from 0, 2, 4, and 6 Gy groups, in monolayer and mammospheres in NSG mice. Data are shown as mean ± SEM and statistical analysis Student’s test to compare IR doses vs 0 Gy (**P < 0.01; *P < 0.05). (B) Representative immunofluorescence images for vimentin of xenograft tumors obtained from mice injected with monolayer and mammosphere cells after 123 days. Original magnification: 20×. Scale bar = 100 μm (C) Quantification of the fluorescence intensities. The average fluorescence intensities were calculated from three parallel immunofluorescence images. Data represent means ± SD (*n* = 3), * P < 0.05 (^#^ *P* < 0.05 for comparison between doses).

Triple‐negative MDA‐MB‐231 irradiated cells grown in monolayer and mammospheres were injected into the mammary gland of female NSG mice and were compared with sham‐irradiated cells (0 Gy; Fig. 2A). Tumors generated by 0 Gy grown in monolayer displayed higher volume than irradiated cells, and tumors emerged 28 days after the injection. In contrast, 6 Gy irradiated cells developed the tumor 58 days after the injection. When we observed tumor size, and evolution, there was a dose‐dependent reduced growth, 6 Gy being the dose that significantly (**P < 0.01) inhibited tumorigenicity (90%). In the case of mammospheres, tumors appeared 28 days after the injection in all groups; however, treatment with 2 and 6 Gy significantly decreased tumor growth, with 6 Gy being the level that generated significantly lower volume tumors (*P < 0.05; Fig. 2A).

These results could suggest that 6 Gy is a dose that negatively affects tumor growth and BCSC formation.

After 123 days, animals were sacrificed and tumors extirpated for immunostaining to detect the expression of the VIMENTIN marker (Fig. 2B) and histological hematoxylin and eosin (Fig. S3). Results showed significantly higher level of VIMENTIN in monolayer cells irradiated at 2 Gy (*P < 0.05). In contrast, in the ALDH1 + mammosphere group, untreated, 2 and 4 Gy irradiated cells showed a high expression of VIMENTIN and 6 Gy irradiated cells displayed a significant decrease (*P < 0.05; Fig. 2B,C).

### 3.4 Effects of IR on selected miRNAs

**Figure 3:**
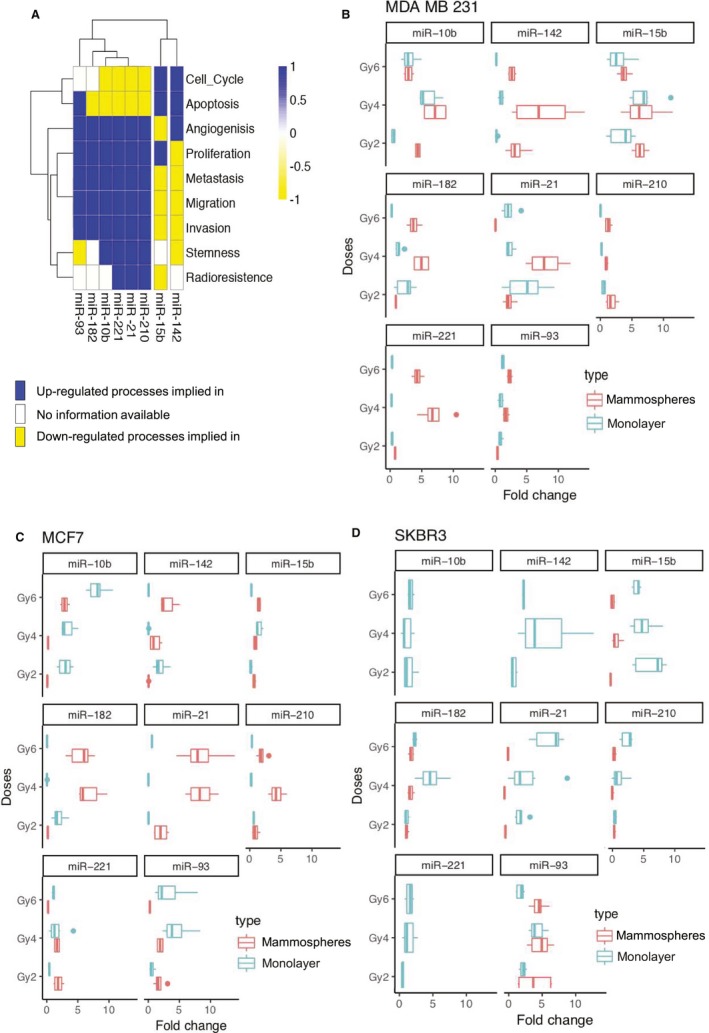
Relative gene expression of selected miRNAs differentially expressed by qRT‐PCR analysis in BC cell lines irradiated vs a 0 Gy sham‐irradiated control. (A) miRNAs heatmap of the biological functions where they are implicated according to the specialized literature using datamining techniques. (B) MDA‐MB‐231 differential expression of miRNAs selected in monolayer and mammospheres. (C) Differential expression of miRNAs in MCF7 monolayer and mammospheres. (D) Differential expression of miRNAs related in SKBR3 cell line. All qRT‐PCR assays were run in triplicate, and data were normalized to 0 Gy and graphed as mean ± SEM. Statistical significance (*P*‐value) of different comparison is represented in Tables 1, 2, 3, 4.

To study the effect of IR on miRNA expression, we selected the following miRNAs, implicated in different tumor processes and stemness properties (Fig. 3A). These miRNAs were differently expressed depending on the tumor cell line studied. In general, MDA‐MB‐231 and MCF7 cells showed a greater miRNA expression in mammospheres than SKBR3 cell line (Fig. 3B–D). In MDA‐MB‐231 mammospheres treated with 4 Gy, miR‐21, miR‐221, miR‐15b, miR‐182, miR‐10b, and miR‐142 were overexpressed in comparison with other doses (Fig. 3B). Monolayer cultures from the same cell line showed a similar expression, and only miR‐142, miR‐210, and miR‐221 displayed lower expression in all the different doses.

When we analyzed MCF7 mammospheres, miR‐21, miR‐142, miR‐182, and miR‐210 were up‐regulated in respect to monolayer culture for the majority of doses, especially for 4 and 6 Gy (Fig. 3C). In contrast, in relation to miR‐10b and miR‐93, we observed a lower expression in mammospheres than in monolayer, where a significant dose‐dependent miRNA expression miR‐15b and miR‐221 showed a low expression in both culture conditions. In contrast, SKBR3 cell line (Fig. 3D) cultured in mammospheres showed a low expression of most miRNAs in all irradiation doses, except for miR‐93 where there was an increased expression for 2 and 4 Gy, and these expressions were similar in monolayer cell cultures. On the other hand, in cells grown in monolayer, miR‐21, miR‐142, miR‐221, miR‐210, and miR‐15b tended to increase more for 4 and 6 Gy. *P* ‐values are described in data (Tables 1, 2, 3, 4).

**Table 1:** Fold changes and *P*‐values corresponding to Fig. 3B. Mann–Whitney *U* nonparametric test was used for comparison between doses.

		*MDA‐MB‐231*					
		Monolayer			Mammospheres ALDH1+		
		2 Gy	4 Gy	6 Gy	2 Gy	4 Gy	6 Gy
miR‐93	Fold	0.83	0.87	1.21	0.37	1.70	2.28
	*P*‐value	0.26	0.34	0.86	**0**.**03**	0.14	**0**.**01**
miR‐10b	Fold	0.52	5.97	3.07	4.39	7.12	2.94
	*P*‐value	0.08	**0**.**00**	**0**.**00**	**0**.**00**	**0**.**00**	**0**.**00**
miR‐15b	Fold	3.43	6.96	2.92	2.92	6.76	3.78
	*P*‐value	**0**.**01**	**0**.**00**	**0**.**04**	**0**.**00**	**0**.**02**	**0**.**00**
miR‐142	Fold	0.22	1.36	0.19	3.40	7.64	2.66
	*P*‐value	0.07	0.36	0.06	**0**.**04**	**0**.**05**	**0**.**00**
miR‐182	Fold	2.40	1.18	0.26	0.91	4.96	1.91
	*P*‐value	**0**.**03**	0.44	**0**.**00**	0.39	**0**.**00**	**0**.**00**
miR‐21	Fold	4.89	2.30	2.20	2.22	8.05	0.05
	*P*‐value	**0**.**00**	**0**.**00**	**0**.**05**	**0**.**05**	**0**.**00**	**0**.**00**
miR‐221	Fold	0.34	0.21	0.32	0.78	7.03	4.28
	*P*‐value	**0**.**00**	**0**.**00**	**0**.**00**	0.09	**0**.**00**	**0**.**00**
miR‐210	Fold	0.53	0.22	0.04	1.76	0.95	1.26
	*P*‐value	0.05	**0**.**00**	**0**.**00**	**0**.**04**	0.56	0.38

Statistical significative *P*‐values are in bold.

**Table 2:** Fold changes and *P*‐values corresponding to Fig. 3C. *U* Mann–Whitney nonparametric test was used for comparison between doses.

		*MCF7*					
		Monolayer			Mammospheres ALDH1+		
		2 Gy	4 Gy	6 Gy	2 Gy	4 Gy	6 Gy
miR‐93	Fold	0.58	4.48	3.31	1.76	1.91	0.25
	*P*‐value	0.11	**0**.**01**	0.09	0.17	**0**.**01**	**0**.**00**
miR‐10b	Fold	2.95	3.32	8.11	0.10	0.22	2.88
	*P*‐value	**0**.**00**	**0.00**	**0.00**	**0.01**	**0.01**	**0.01**
miR‐15b	Fold	0.18	1.48	0.26	0.70	0.90	1.46
	*P*‐value	**0.00**	0.06	**0.00**	**0.02**	0.06	**0.01**
miR‐142	Fold	1.91	0.03	3.05	0.05	1.08	2.85
	*P*‐value	0.49	**0.01**	0.15	**0.00**	0.84	**0.00**
miR‐182	Fold	1.96	0.03	0.08	0.18	6.75	5.50
	*P*‐value	0.08	**0.00**	**0.00**	**0.00**	**0.00**	**0.00**
miR‐21	Fold	0.04	0.02	0.57	2.04	8.45	8.57
	*P*‐value	**0.00**	**0.00**	**0.00**	0.26	**0.00**	**0.01**
miR‐221	Fold	0.43	1.64	1.09	1.87	1.65	0.22
	*P*‐value	**0.01**	0.43	0.87	0.07	**0.03**	**0.00**
miR‐210	Fold	0.63	0.25	0.36	0.92	4.34	1.91
	*P*‐value	**0.02**	**0.00**	**0.00**	0.65	**0.00**	0.18

Statistical significative *P*‐values are in bold.

**Table 3:** Fold changes and *P*‐values corresponding to Fig. 3D. *U* Mann–Whitney nonparametric test was used for comparison between doses.

		*SKBR3*					
		Monolayer			Mammospheres ALDH1+		
		2 Gy	4 Gy	6 Gy	2 Gy	4 Gy	6 Gy
miR‐93	Fold	1.21	2.13	1.06	0.31	2.34	2.25
	*P*‐value	0.11	**0.00**	0.80	0.06	**0.00**	**0.00**
miR‐10b	Fold	0.59	0.48	0.73			
	*P*‐value	**0.03**	**0.03**	0.11			
miR‐15b	Fold	2.77	2.15	1.81	0.08	0.44	0.20
	*P*‐value	**0.00**	**0.00**	**0.00**	**0.01**	**0.00**	**0.00**
miR‐142	Fold	0.59	2.62	1.25			
	*P*‐value	**0.00**	**0.04**	**0.00**			
miR‐182	Fold	0.46	2.02	1.01	0.48	0.73	0.76
	*P*‐value	**0.00**	**0.01**	0.85	0.07	0.08	0.25
miR‐21	Fold	1.26	1.51	2.92	0.07	0.00	0.23
	*P*‐value	0.50	0.57	**0.01**	**0.00**	**0.00**	**0.00**
miR‐221	Fold	0.22	0.62	0.66			
	*P*‐value	0.20	0.05	**0.03**			
miR‐210	Fold	0.32	0.61	1.16	0.31	0.19	0.31
	*P*‐value	**0.00**	0.31	0.30	**0.00**	**0.00**	**0.00**

Statistical significative *P*‐values are in bold.

**Table 4:** *P*‐values comparing monolayer and ALDH1 + mammospheres fold changes. Kruskal–Wallis nonparametric tests were used.

	MDA‐MB‐231			MCF7			SKBR3		
	2 Gy	4 Gy	6 Gy	2 Gy	4 Gy	6 Gy	2 Gy	4 Gy	6 Gy
miR‐93	**0.01**	**0.03**	**0.01**	**0.03**	**0.03**	**0.01**	0.81	0.57	**0.00**
miR‐10b	**0.01**	0.09	0.83	**0.01**	**0.01**	**0.01**			
miR‐15b	**0.03**	1.00	0.39	0.07	0.06	**0.01**	**0.00**	**0.00**	**0.00**
miR‐142	**0.01**	**0.01**	**0.01**	**0.01**	**0.00**	0.06			
miR‐182	0.39	**0.01**	**0.01**	**0.00**	**0.00**	**0.00**	0.81	**0.00**	**0.02**
miR‐21	0.29	**0.01**	**0.01**	**0.01**	**0.01**	**0.01**	**0.00**	**0.00**	**0.00**
miR‐221	**0.01**	**0.01**	**0.01**	**0.01**	0.67	**0.01**			
miR‐210	**0.01**	**0.00**	**0.00**	0.67	**0.01**	**0.01**	0.97	**0.02**	**0.00**

Statistical significative *P*‐values are in bold.

### 3.5 Expression of selected miRNAs in breast cancer patients treated with RT

**Figure 4:**
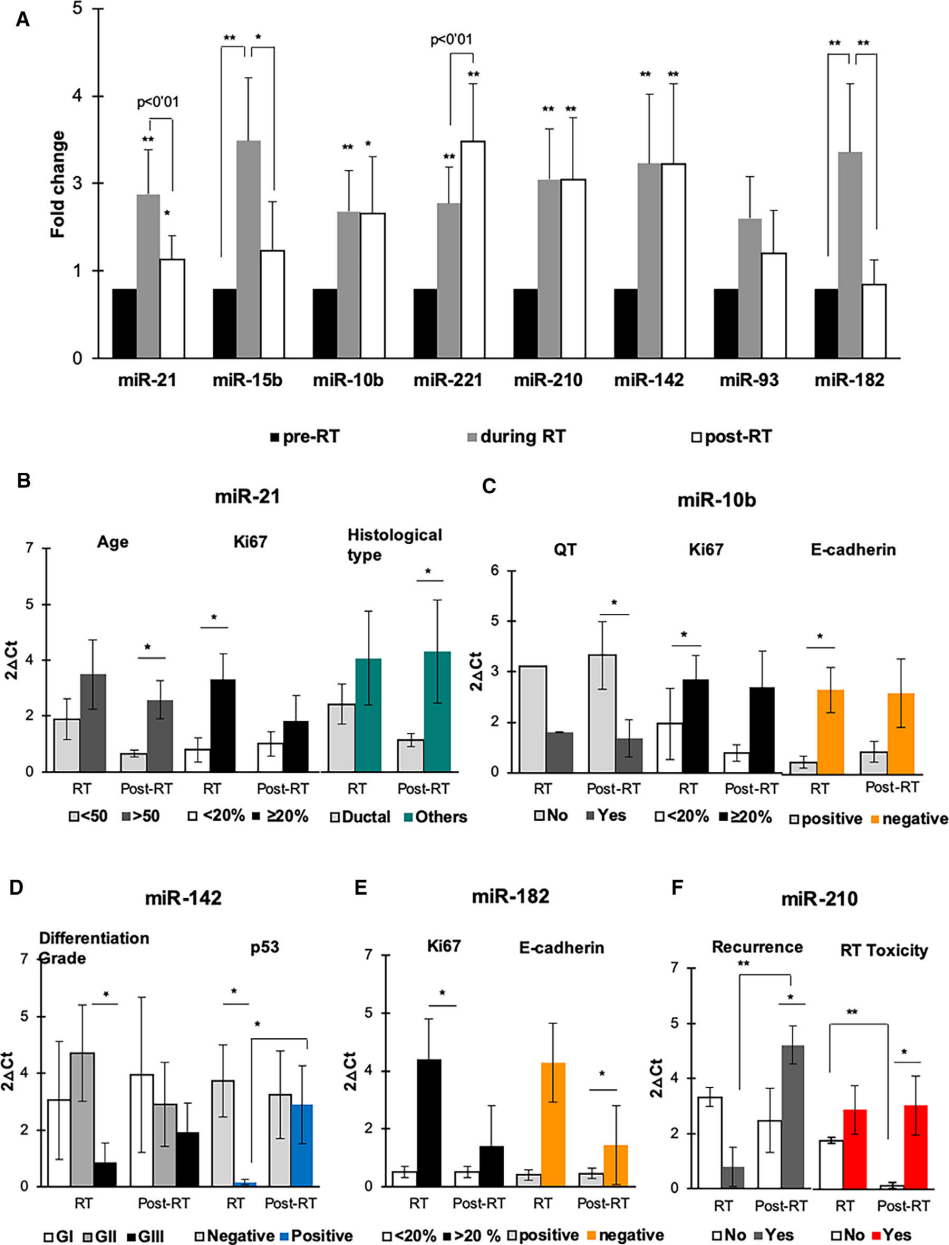
Relative gene expression of selected miRNAs differentially expressed by qRT‐PCR analysis in BC patients treated with RT vs pretreatment samples. (A) miRNA expression levels pre‐RT (control), during RT, and post‐RT. (B–F) Representation of significant miRNA expression changes when aggregated by clinicopathological features. Data are mean values ± SEM. *P < 0.05 and **P < 0.01 show the significant values calculated using *t‐*test and Kruskal–Wallis test. See Table S3 for *P*‐values.

To examine the modulation of miRNAs in patient’s serum, Fig. 4A shows the differential expression of miRNAs compared to pre‐RT, during RT, and post‐RT. We observed that all miRNA expression significantly increased during RT (**P < 0.01) except miR‐93. In addition, in comparison with pre‐RT, miR‐21 and miR‐10b expression increased in post‐RT (*P < 0.05), and very significantly (**P < 0.01) for miR‐221, miR‐210, and miR‐142. When compared, during RT and post‐RT groups had significant differences, with a decrease of expression of miR‐21, mir‐15b, and miR‐182, and an increased expression of miR‐221. Also, in Fig. S4 the fold change of miRNA expression in the three TNBC patients is shown (*P < 0.05, **P < 0.01).

When grouped by the clinicopathological characteristics of the patients (age, menopausal status, tumor classification, Ki67, etc.) that are described in Table 5, we observed that miR‐21 (Fig. 4B) was significant for age and histological type in post‐RT and for Ki67 during RT; miR‐10b (Fig. 4C) showed significance during RT for Ki67 and E‐CADHERIN, but when grouped by chemotherapy in post‐RT; miR‐142 (Fig. 4D) for differentiation grade (GII vs GIII) and marker p53 in treatment; in miR‐182 (Fig. 4E) also found significance for Ki67 and E‐CADHERIN during RT and finally miR‐210 (Fig. 4F) showed to be significant with recurrence and toxicity after RT (Table S3).

**Table 5:** Clinicopathological features of BC patients studied. In each variable, the population (*n*) was shown with respect to the total population.

Variables	*N* = 20	Recurrence	
		No	Yes
Age			
< 50	10	9	1
> 50	10	8	2
Menopausal status			
Premenopausal	10	9	1
Menopausal	6	5	1
Postmenopausal	4	3	1
Tumor classification			
Triple negative	3	2	1
ER+/PR+	17	15	2
Differentiation grade			
G I	9	8	1
G II	7	6	1
G III	4	3	1
Histological type			
Ductal	17	15	2
Other	3	2	1
E‐*CADHERIN*			
Positive	16	14	2
Negative	4	3	1
p53			
Positive	3	2	1
Negative	17	15	2
Ki67			
< 20%	14	13	1
≥ 20%	6	4	2
Chemotherapy			
No QT	9	8	1
Yes QT	11	9	2
Radiation doses			
2 Gy	7	6	1
2.65 Gy	13	11	2
RT toxicity			
Yes	18	15	3
No	2	2	0
Recurrence (end of trial)			
Yes	3		
No	17		

## 4 Discussion

In our study, we found how different doses of IR induce the selection of BC cells (MDA‐MB‐231, MCF7 and SKBR3) with stemness properties and how these doses modify the expression levels of miRNAs related to important oncogenic processes in BC. For years, several studies have demonstrated that IR enhances BCSC‐like phenotype (Gao *et al.*, 2016; Gomez‐Casal *et al.*, 2013; Kim *et al.*, 2015). Different to these previous works, we analyze the effect of IR on the three most common BC molecular subtypes (luminal, HER2+, and TNBC). Interestingly, our results showed that, in both MCF7 and SKBR3 mammospheres, all IR doses decreased ALDH1 activity and CD44^+^/CD24^−/low^ expression. In contrast, triple‐negative BCSCs in all IR doses significantly increased the expression of CD44^+^/CD24^−/low^ surface markers, which has been related to radioresistance and poor prognosis in BC patients (Kim *et al.*, 2016; Phillips *et al.*, 2006; Wang *et al.*, 2017). In the same way, pluripotency (Kim *et al.*, 2018; Takahashi and Yamanaka, 2006) and EMT‐related gene (Theys *et al.*, 2016; Zhou *et al.*, 2011) differences observed for each cell line after treatment can be explained by the different sensitivity of BC molecular subtypes to IR (Kim *et al.*, 2015).

For the *in vivo* tumorigenic capacity after IR, the MDA‐MB‐231 TNBC cell line was chosen because of its described increased stemness properties, the potent migratory response, and aggressiveness in mice (Price *et al.*, 1999). Our results support that IR negatively affects tumor growth when increased doses in cells are cultured in monolayer; however, in mammospheres animal model, and according to the *in vitro* results, we observed a similar growth rate in sham‐irradiated controls and the 4 Gy group. This suggests that 4 Gy selected the more resistant triple‐negative BCSCs *in vitro*, which had more aggressive behavior *in vivo*. In fact, the great heterogeneity of BCSC (Da Cruz Paula and Lopes, 2017; Hernández‐Camarero *et al.*, 2018) has been reported and that CSC plasticity may be a common response to IR with the generation of new induced BCSCs resistant to specific IR doses, specifically SUM159PT ALDH‐ triple‐negative BCSCs tumors irradiated with 4Gy, induced more aggressive BCSC subpopulations (Lagadec *et al.*, 2012). In addition, high levels of VIMENTIN, an indicator of BC progression (Calaf *et al.*, 2014), were found in tumors derived from monolayer cultures irradiated with 2 Gy, and those derived by mammospheres irradiated with 4 Gy showing concordance with EMT‐gene expression found *in vitro*.

Nevertheless, the main purpose of our work was to analyze the role of determined key miRNAs (Summerer *et al.*, 2013) in response to IR in both CSC‐like cells and BC patients that could be useful at the clinical level. We studied miR‐21 and miR‐182, as recognized oncogenic miRNAs that promote cell proliferation and metastasis, and are valuable markers of prognosis in BC (Shah and Chen, 2014). We observed a different behavior in their response to IR, being highly expressed after treatment with 4 and 6 Gy in both MDA‐MB‐231 and MCF7 mammospheres. It has been demonstrated that miR‐21 is up‐regulated and contributes to IR resistance upon high doses of irradiation (5Gy) in BC cells, since this miRNA influences cell cycle progression *via* the DNA damage‐G2 checkpoint induction (Anastasov *et al.*, 2012). Equally, the overexpression of miR‐182 confers radioresistance in non‐small‐cell lung cancer (Chen *et al.*, 2019). In addition, mir‐26b increased in radiation‐associated BC in female post‐Chernobyl clean‐up workers in comparison with nonexposed control (Wilke *et al.*, 2018). These miRNAs related to radiation exposure associated with DNA damage response and tumor progression could represent radiation markers in BC.

In our analysis, miRNAs related to metastasis, invasion, and CSCs, such as miR‐221, miR‐10b, and miR‐93, were analyzed with miR‐221 being the only one that increased after treatment with 4 and 6 Gy in MDA‐MB‐231 mammospheres. It is known that miR‐221 induces expression of pluripotency‐associated genes, enforcing stemness phenotype, mammosphere formation, and radioresistance processes (Roscigno *et al.*, 2016; Zhang *et al.*, 2011). On the other hand, miR‐93 and miR‐10b overexpression are related to cancer development and metastatic BC progression (Korpela *et al.*, 2015; Li *et al.*, 2017; Ma, 2010). In fact, we observed an increased expression of miR‐93 in SKBR3 mammospheres at 4 and 6 Gy, and in MDA‐MB‐231 mammospheres at 2 Gy. Moreover, miR‐10b was mainly overexpressed for all doses in TNBC. These results suggest that BCSC subpopulations with a more aggressive behavior were selected after high IR for Her2 + CSCs and TNBCSCs, in contrast to HR + BCSCs, where high doses significantly decreased the expression of those miRNAs. Therefore, these findings indicate that IR was effective against HR + BCSCs and that miR221, miR‐93, and miR‐10b could be useful markers for IR response in BC patients.

The miR‐210, which was overexpressed in MDA‐MB 231 mammospheres and mainly in MCF7 mammospheres at high doses of radiation, was found to stabilize the hypoxia‐inducible factor‐1 and enhance radioresistance *in vitro* (Wilson and Hay, 2011). In fact, a recent study showed that the hypoxic microenvironment maintains CSC phenotype, which may influence their intrinsic resistance to radiation (Korpela *et al.*, 2015). Besides, miR‐142, which downregulates BCSC phenotype and decreases radioresistance *in vitro* (Troschel *et al.*, 2018), and miR‐15b that belongs to the miR‐15 family related to BC cell radiosensitivity by influencing G 2/M checkpoint proteins (Mei *et al.*, 2015) increased at 6 Gy in MCF‐7‐BCSCs. In agreement with these results, several miRNAs such as miR‐139, miR‐125b, and mir‐223 considered as tumor suppressors and that increase after IR are related to radiosensitivity and as markers of response to RT (Fabris *et al.*, 2016; Metheetrairut *et al.*, 2017; Pajic *et al.*, 2018).

Finally, we grouped the patients by their clinicopathological characteristics and compared them with miRNA expression. Thereby, our results displayed that miR‐21, miR‐182, and miR‐10b were significantly increased during RT period in patients who were positive for Ki67, an indicator of proliferation, whose high expression has been related to worse prognosis, recurrence, and death in BC (Yerushalmi *et al.*, 2010). miR‐10b and miR‐182 were also overexpressed in patients negative for E‐Cadherin during RT, whose positive expression is correlated to a better prognosis and survival (Yang *et al.*, 2018). Moreover, miR‐142, that is related to radiosensitivity and acts as a tumor suppressor in HR + BC (Mansoori *et al.*, 2019), was downregulated in patients with Grade III of differentiation and p53 positive. Finally, miR‐210, which is related to poor prognosis and metastasis (Hong *et al.*, 2012), was overexpressed in patients with relapse after treatment and was also overexpressed in patients showing toxicity after RT. In addition, there was also concordance between the increased expression of both miR‐210 and miR‐221 in TNBC mammospheres and the levels found in the only TNBC patient p53 + that had recurrence after RT (Friedrichs *et al.*, 1993).

## 5 Conclusions

Despite the rapid expansion of using miRNAs as possible biomarkers, however, there are not many clinical studies of miRNAs with clinical utility in RT. In this sense, our study supports how miRNAs related to BCSC subpopulations could provide a useful method to predict and monitor tumor radio‐response depending on the molecular BC subtype. Further studies including an elevated number of BC patients treated with RT should be done to have more robust results useful in the clinic. Nonetheless, future clinical implementation of miRNA signature determination as a liquid biopsy, for personalized and precision RT dosage regimes, is necessary to improve prognosis, treatments, and survival of BC patients.

## Conflict of interest

The authors declare no conflict of interest.

## Author contributions

CG‐L conceived and designed the study; collected, assembled, and analyzed the data; and interpreted and wrote the manuscript. MAO‐U assembled, analyzed, and interpreted the data. GJ, EL‐R, and HB analyzed the data and interpreted and wrote the manuscript. CdV, CM‐T, JME, and ARG‐R analyzed and interpreted the data. MZH assembled and analyzed the data. MIN conceived and designed the study; analyzed and interpreted the data; wrote the manuscript; and gave final approval of the manuscript and financial support. JAM conceived and designed the study; analyzed and interpreted the data; gave financial and administrative support; provided the study materials; and wrote the manuscript and gave final approval of the manuscript.

## Supporting information


**Fig. S1.** Differential expression of pluripotency and EMT genes mRNA of breast cancer cell monolayer and mammospheres cultures.
**Fig. S2.** (A) Representative images of mammospheres formed from different IR doses in MDA‐MB‐231 and SKBR3. Scale bar = 100 m. (B) Representative images of colonies formed from different IR doses in MDA‐MB‐231 and SKBR3.
**Fig. S3.** Representative images of hematoxylin/eosin staining of TNBC line (MDA‐MB 231) obtained from 0, 2, 4 and 6 Gy mice tumors.
**Fig. S4.** miRNA expression levels pre‐RT, during RT and post‐RT of triple‐negative breast cancer patients.
**Table S1.** Primer sequences used to qRT‐PCR for mRNA.
**Table S2.** Primer sequences used to qRT‐PCR for miRNA.
**Table S3.** Statistical data of P‐values corresponding to Fig. 4B–F.
