# Supplementary material for: miRNAs as radio‐response biomarkers for breast cancer stem cells
Source: Mol Oncol. 2020 Feb 6;14(3):556–70. doi: 10.1002/1878-0261.12635 (PMC7053246; doi:10.1002/1878-0261.12635)
Supplement: Supplementary file 1 — Fig. S1. Differential expression of pluripotency and EMT genes mRNA of breast cancer cell monolayer and mammospheres cultures. Fig. S2. (A) Representative images of mammospheres formed from different IR doses in MDA‐MB‐231 and SKBR3. Scale bar = 100 m. (B) Representative images of colonies formed from different IR doses in MDA‐MB‐231 and SKBR3. Fig. S3. Representative images of hematoxylin/eosin staining of TNBC line (MDA‐MB 231) obtained from 0, 2, 4 and 6 Gy mice tumors. Fig. S4. miRNA expression levels pre‐RT, during RT and post‐RT of triple‐negative breast cancer patients. Table S1. Primer sequences used to qRT‐PCR for mRNA. Table S2. Primer sequences used to qRT‐PCR for miRNA. Table S3. Statistical data of P‐values corresponding to Fig. 4B–F. [file MOL2-14-556-s001.docx]

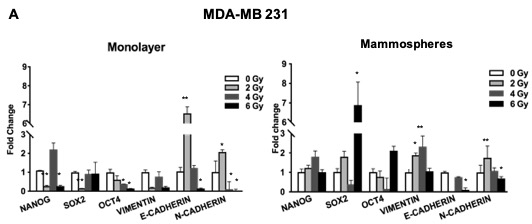

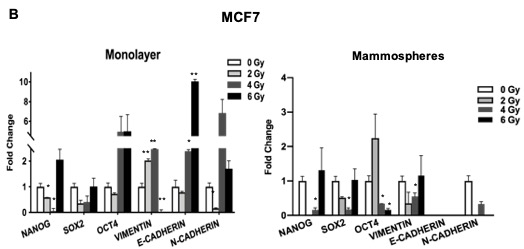

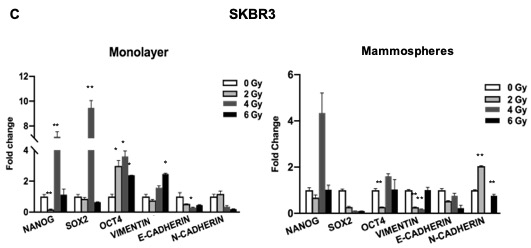
**Figure S1.** Differential expression of pluripotency and EMT genes mRNA of breast cancer cell monolayer and mammospheres cultures. qRT-PCR analysis for the expression of monolayer and mammospheres of pluripotency and EMT-related genes for 0 Gy, 2 Gy, 4 Gy and 6 Gy in MDA-MB-231 (A), MCF7 (B) and SKBR3 (C) cell lines. The statistical comparison was 0 Gy versus 2, 4 and 6 Gy. Data are normalized to 1 for 0 Gy using GAPDH as internal control, and graphed as mean ± SEM **p < 0.05* and ***p < 0.01*.

**
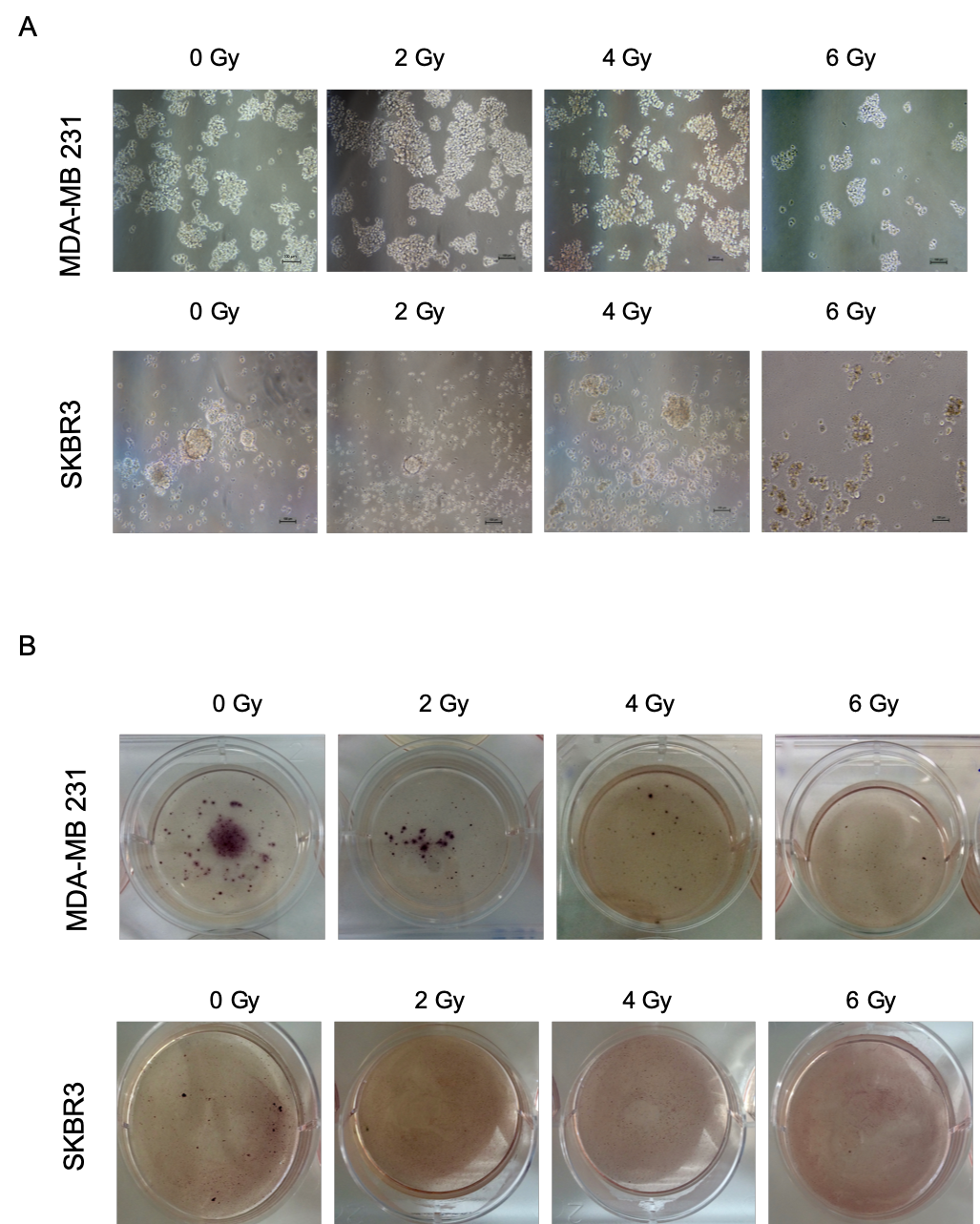
 Figure S2.** (A) Representative images of mammospheres formed from different IR doses in MDA-MB-231 and SKBR3. Scale bar = 100 μm. (B) Representative images of colonies formed from different IR doses in MDA-MB-231 and SKBR3.


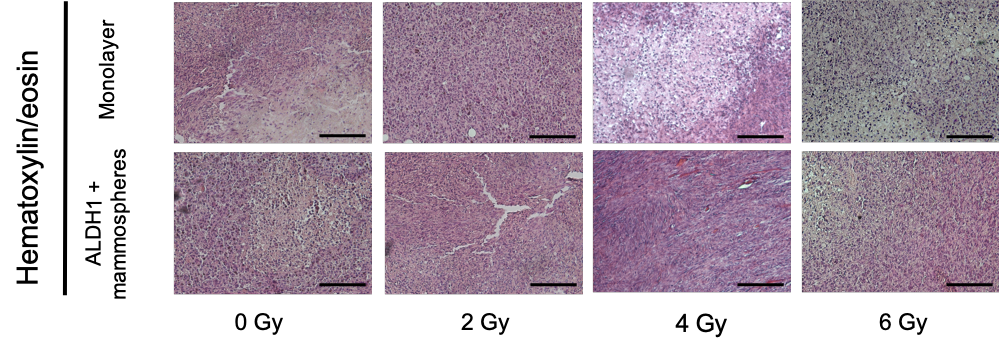


**Figure S3.** Representative images of hematoxylin/eosin staining of TNBC line (MDA-MB-231) obtained from 0, 2, 4 and 6 Gy mice tumors. Original magnification: 20X. Scale bar = 100 μm.


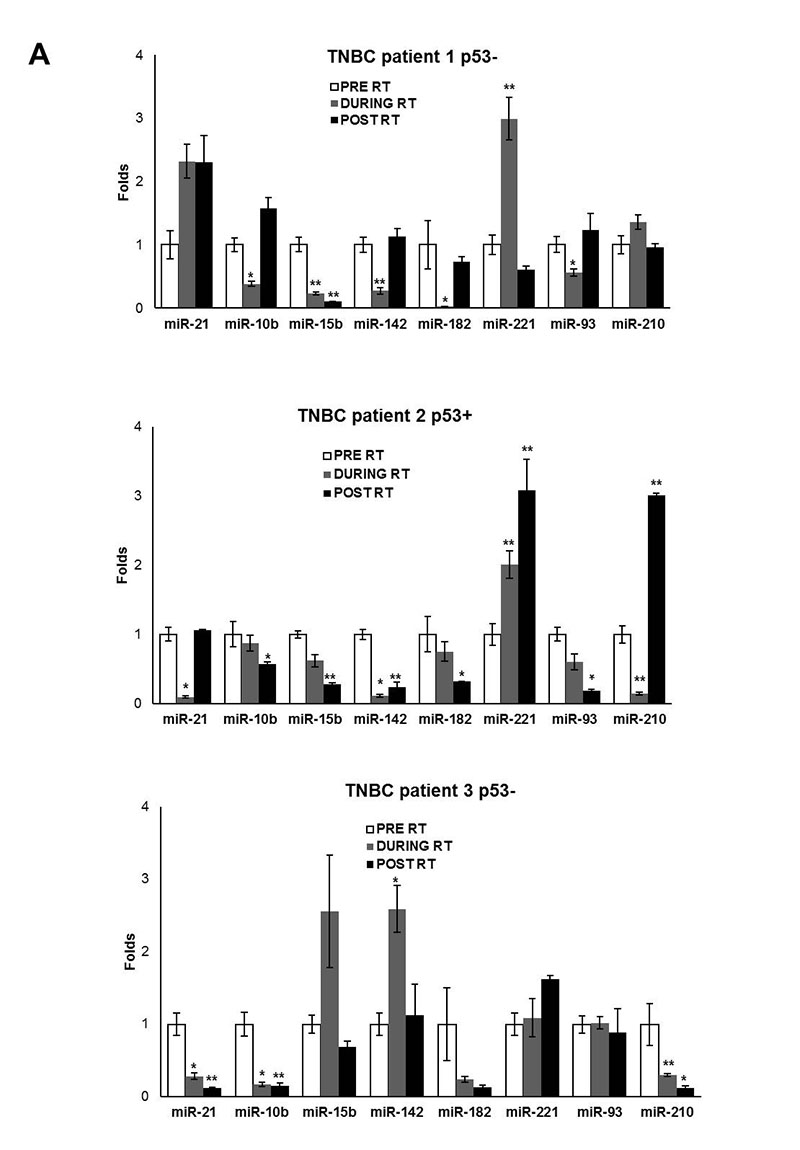


**Figure S4.** miRNA expression levels pre-RT, during-RT and post-RT of triple-negative breast cancer patients. Data are mean value ± SEM. *p<0.05 and **p<0.01.

**TABLES:**

**Supplementary Table S1.** Primer sequences used to qRT-PCR for mRNA

| **Gene** | **Primer Sequence** | |
| --- | --- | --- |
| ***NANOG*** | Forward | 5´ TCCTGAACCTCAGCTACAAAC 3´ |
|  | Reverse | 5´ GCGTCACACCATTGCTATTC 3´ |
| ***SOX2*** | Forward | 5´ GGAGCTTTGCAGGAAGTTTG 3´ |
|  | Reverse | 5´ GGAAAGTTGGGATCGAACAA 3´ |
| ***OCT4*** | Forward | 5´ CACCATCTGTCGCTTCGAGG 3´ |
|  | Reverse | 5´ AGGGTCTCCGATTGCATATCT 3´ |
| ***E-CADHERIN*** | Forward | 5´ AATTCCTGCCATTCTGGGGA 3´ |
|  | Reverse | 5´ TCTTCTCCGCCTCCTTCTTC 3´ |
| ***N-CADHERIN*** | Forward | 5´ TGAGCCTGAAGCCAACCTTA 3´ |
|  | Reverse | 5´ AGGTCCCCTGGAGTTTTCTG 3´ |
| ***VIMENTIN*** | Forward | 5´ AGCTAACCAACGACAAAGCC 3´ |
|  | Reverse | 5´ TCCACTTTGCGTTCAAGGTC 3´ |

**Supplementary Table S2.** Primer sequences used to qRT-PCR for miRNA

| **miRNA** | Mature sequence |
| --- | --- |
| **hsa-miR-210-3p** | CUGUGCGUGUGACAGCGGCUGA |
| **hsa-miR-10b-5p** | UACCCUGUAGAACCGAAUUUGUG |
| **hsa-miR-182-3p** | UGGUUCUAGACUUGCCAACUA |
| **hsa-miR-142-3p** | UGUAGUGUUUCCUACUUUAUGGA |
| **hsa-miR-221-3p** | AGCUACAUUGUCUGCUGGGUUUC |
| **hsa-miR-21-3p** | CAACACCAGUCGAUGGGCUGU |
| **hsa-miR-93-5p** | CAAAGUGCUGUUCGUGCAGGUAG |
| **hsa-miR-15b-5p** | UAGCAGCACAUCAUGGUUUACA |
| **hsa-miR-24-3p** | UGGCUCAGUUCAGCAGGAACAG |
| **hsa-miR-425-5p** | AAUGACACGAUCACUCCCGUUGA |

**Supplementary table S3** Statistical data of p-values corresponding to Figure 4 B;C;D;E;F

| **Groups** | | **RT** | **Post- RT** |  |
| --- | --- | --- | --- | --- |
|  |  | **p-value** | **p-value** |  |
| **miR-21** | | | | |
| **Age** | **< 50** | 0,43 | **0,04** |  |
|  | **> 50** |  |  |  |
| **Histological**  **type** | **Ductal** | 0,31 | **0,02** |  |
|  | **Others** |  |  |  |
| **Ki67** | **< 20%** | **0,05** | 0,275 |  |
|  | **≥ 20%** |  |  |  |
| **miR-10b** | | | | |
| **Chemotherapy** | **no** | **0,04** | 0,09 |  |
|  | **yes** |  |  |  |
| **Ki67** | **< 20%** | **0,05** | 0,266 |  |
|  | **≥ 20%** |  |  |  |
| **E-cadherin** | **positive** | **0,038** | 0,549 |  |
|  | **negative** |  |  |  |
| **miR-142** | | | | |
| **p53** | **positive** | **0,04** | 0,74 |  |
|  | **negative** |  |  |  |
| **Histological**  **grade** | **GI vs GII** | 0,053 | 0,72 |  |
|  | **GI vs GIII** | 0,28 | 0,22 |  |
|  | **GII vs GIII** | **0,019** | 0,34 |  |
| **miR-182** | | | | |
| **Ki67** | **< 20%** | **0,04** | 0,57 |  |
|  | **≥ 20%** |  |  |  |
| **E-cadherin** | **positive** | **0,046** | 0,277 |  |
|  | **negative** |  |  |  |
| **miR-210** | | | | |
| **Recurrence** | **no** | 0,35 | **0,03** |  |
|  | **yes** |  |  |  |
| **Toxicity** | **No** | 0,69 | **0,046** |  |
|  | **yes** |  |  |  |
